# Dysregulated CD46 shedding interferes with Th1‐contraction in systemic lupus erythematosus

**DOI:** 10.1002/eji.201646822

**Published:** 2017-05-22

**Authors:** Ursula Ellinghaus, Andrea Cortini, Christopher L. Pinder, Gaelle Le Friec, Claudia Kemper, Timothy J. Vyse

**Affiliations:** ^1^ Division of Genetics and Molecular Medicine Department of Medical and Molecular Genetics King's College London Guy's Hospital London UK; ^2^ MRC Centre for Transplantation Division of Transplant Immunology and Mucosal Biology King's College London UK; ^3^ Laboratory of Molecular Immunology and the Immunology Center National Heart, Lung, and Blood Institute (NHLBI) National Institutes of Health (NIH) Bethesda MD USA

**Keywords:** Autoimmunity, CD46, Complement, Innate immunity, Membrane cofactor protein, T cell, Th1

## Abstract

IFN‐γ‐producing T helper 1 (Th1) cell responses mediate protection against infections but uncontrolled Th1 activity also contributes to a broad range of autoimmune diseases. Autocrine complement activation has recently emerged as key in the induction and contraction of human Th1 immunity: activation of the complement regulator CD46 and the C3aR expressed by CD4^+^ T cells via autocrine generated ligands C3b and C3a, respectively, are critical to IFN‐γ production. Further, CD46‐mediated signals also induce co‐expression of immunosuppressive IL‐10 in Th1 cells and transition into a (self)‐regulating and contracting phase. In consequence, C3 or CD46‐deficient patients suffer from recurrent infections while dysregulation of CD46 signaling contributes to Th1 hyperactivity in rheumatoid arthritis and multiple sclerosis. Here, we report a defect in CD46‐regulated Th1 contraction in patients with systemic lupus erythematosus (SLE). We observed that MMP‐9‐mediated increased shedding of soluble CD46 by Th1 cells was associated with this defect and that inhibition of MMP‐9 activity normalized release of soluble CD46 and restored Th1 contraction in patients’ T cells. These data may deliver the first mechanistic explanation for the increased serum CD46 levels observed in SLE patients and indicate that targeting CD46‐cleaving proteases could be a novel avenue to modulate Th1 responses.

## Introduction

Systemic lupus erythematosus (SLE) is a complex autoimmune disease of uncertain etiology 1. One hallmark of SLE is dysregulated cytokine production, which contributes to immune dysfunction and tissue inflammation 2. Type I and II interferons (IFN) 3 have been implicated in SLE induction and progression. Although much emphasis has been placed on type I IFNs 4, numerous reports indicate that increased levels of the single type II interferon, IFN‐gamma (IFN‐γ), is required in both spontaneous and induced animal models of SLE 5, 6.

CD4^+^ T helper type 1 (Th1) lymphocytes are a major source of IFN‐γ 7, 8. In humans, additional signals (aside from CD28), mediated by the complement regulator CD46 have recently emerged as crucial to Th1 induction 9, 10, 11. CD46 was initially discovered as a complement regulatory protein functioning: a cell‐surface cofactor mediating cleavage of C3b and C4b 12. Interestingly, engagement of CD46 on TCR‐stimulated CD4^+^ T cells seems largely independent of serum‐derived C3b, rather T cell CD46 stimulation is mediated by T‐cell‐derived C3b (generated intra‐ and extra‐cellularly via cathepsin L‐induced cleavage of C3). Such autocrine C3b‐driven CD46 engagement delivers signals to the T cells that induce the controlled regulation of IL‐2 receptor (IL‐2R) expression 10 and reprogramming specifically needed for IFN‐γ secretion 11. Furthermore, while lack of CD46 co‐stimulation during T‐cell activation (in patients with C3 or CD46 deficiency) leads to severely reduced Th1 responses and recurrent infections 10, 11, 13, pathologically increased intracellular C3 activation and autocrine CD46 simulation is connected with the hyperactive Th1 responses present in rheumatoid arthritis (RA) 14.

CD46‐mediated signals are not only required for successful Th1 induction (IFN‐γ^+^ cells), they also contribute to Th1 contraction. CD46 engagement, together with currently undefined signals mediated by the IL‐2R, leads to IL‐10 co‐expression in Th1 cells and the induction of a (self)‐regulatory (IFN‐γ^+^ IL‐10^+^ T cell) and finally contracting phase with IL‐10^+^ Th1 cells 9, 15. Dysregulated CD46 signals during the contracting phase prevent normal IFN‐γ shut‐down and the switch into normal Th1 contraction and contribute to disease pathology in RA and multiple sclerosis (MS) 15, 16.

Thus, cell surface expression of CD46 is tightly regulated in various cell types and it has long been known that the extracellular portion of CD46 is rapidly down‐regulated post CD46 activation 17. CD46's ‘disappearance’ is due to both the regulated internalization of the molecule 17 and to CD46 shedding via matrix metalloproteinases (MMPs) 18. Although the functional significance of CD46 shedding is not fully understood, we recently demonstrated that CD46 expression on resting T cells contributes to T‐cell homeostasis as CD46 interacts directly with Jagged1 and prevents a Notch1‐Jagged1 interaction that would normally promote T‐cell activation 10. TCR‐induced C3b generation and autocrine CD46 engagement leads to shedding of CD46 (with the CD46‐cleaving protease not yet defined), which then allows a productive Notch1‐Jagged1 interaction required for normal human Th1 induction 19. Thus, CD46 surface expression on non‐stimulated T cells acts as a ‘stop signal’, but provides a ‘go signal’ when immune activation is apparent. Importantly, soluble CD46 (sCD46) generated during normal T‐cell activation can also modulate Notch1‐mediated signals on T cells and impacts significantly on IFN‐γ to IL‐10 switching and Th1 contraction 10. Intriguingly, increased levels of sCD46 have been reported in sera of patients with RA, MS, and SLE 20, 21. Together, these observations prompted us to investigate expression and processing of CD46 as well as the CD46‐driven Th1 induction and contraction ‘states’ of CD4^+^ T cells from patients with SLE in vitro.

## Results

### Th1 contraction is defective in CD4^+^ T cells from patients with SLE

**Figure 1:**
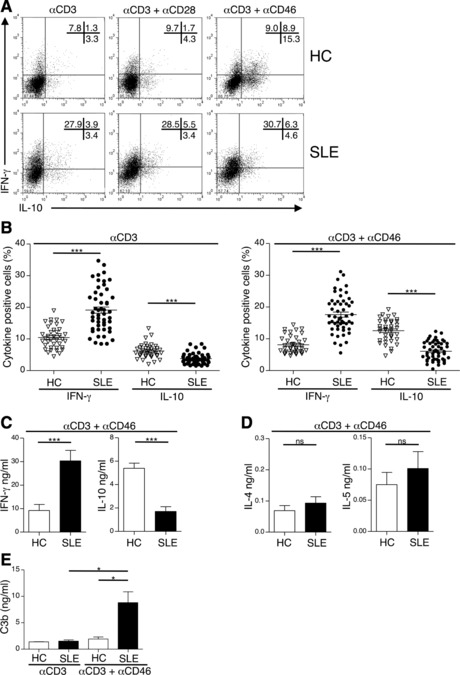
CD46‐regulated Th1 contraction (IFN‐γ to IL‐10 switching) is defective in Th1 cells from patients with SLE. (A) Active cytokine secretion at 36 h by purified CD4^+^ T cells from healthy controls (HC, upper row) or SLE patients (lower row) not activated or activated with mAbs to CD3 alone or to CD3 and CD46. Numbers in plots indicate percent IFN‐γ^+^IL‐10^−^ cells (top left), IFN‐γ^+^IL‐10^+^ cells (top right) or IFN‐γ^−^IL‐10^+^ cells (bottom right). One representative FACS analysis of 38 independent healthy controls is shown. (B) Summary of altered cytokine secretion of CD3, and CD3+ CD46‐activated CD4^+^ T cells of patients with SLE. Experiments were performed as under (A) with CD4^+^ T cells of HCs (*n* = 38) and patients with SLE (*n* = 45) and cumulative data of 35 independent experiments are shown. Data are plotted as percentages of either total numbers of IFN‐γ‐positive (IFN‐γ^+^IL‐10^−^ and IFN‐γ^+^IL‐10^+^) or IL‐10‐positive (IFN‐γ^+^IL‐10^+^ and IFN‐γ^−^IL‐10^+^) cells. (C and D) IFN‐γ, IL‐10 (C), IL‐4 and IL‐5 (D) accumulation in cell supernatants of CD3^+^CD46‐activated T cells from HCs and SLE patients 36 h post activation. Data are shown as mean ± SD and are representative of 20 experiments using 10 HCs and 10 SLE patients. (E) Increased production of the CD46 ligand C3b by T cells from SLE patients. Purified T cells were activated for 36 h as indicated and C3b generation and secretion measured by ELISA. Data are shown as mean ± SD derived from experiments using 8 HCs and 10 SLE patients. *p* values in (B‐D) were calculated using the Mann–Whitney U test. *p < 0.05; ***p < 0.0001.

Initial signals through CD46 are required for Th1 induction and IFN‐γ secretion; while later, CD46 induces co‐production of immunosuppressive IL‐10 and Th1 contraction 9, 15. To assess if perturbation in the complement‐CD46‐driven pathways contribute to dysregulated Th1 responses observed in SLE, we activated this pathway in purified CD4^+^ T cells from healthy controls (HCs) and individuals with SLE (see Methods) and measured active IFN‐γ and IL‐10 secretion. SLE CD4^+^ T cells showed a statistically significant increase in IFN‐γ‐positive T cells when compared to HC T cells following CD3 activation and, furthermore, failed to switch efficiently from IFN‐γ to IL‐10 production following CD3+CD46 stimulation (Fig. 1A and B). In addition, SLE T cells secreted significantly higher (up to 300%) amounts of IFN‐γ while IL‐10 levels were substantially reduced (up to 300%) after CD3+CD46 stimulation (Fig. 1C). This shifted the IFN‐γ:IL‐10 ratio significantly toward IFN‐γ in SLE compared to controls. Importantly, CD46‐mediated signals were specifically required for normal Th1, but not Th2 responses, because the IL‐4 and IL‐5 levels were unaltered in SLE T cells after CD3+CD46 activation (Fig. 1D) or after CD3 and CD3+CD28 stimulation (not shown). Recent work demonstrates that complement C3 can be cleaved intracellularly by cathepsin L into C3a/C3b by human CD4^+^ T cells 14. TCR activation translocates these stored activation products rapidly to the cell surface were C3b engages CD46 to induce IFN‐γ. Interestingly, CD3+CD46‐activated T cells from SLE patients secrete significantly higher levels of C3b compared to HCs (Fig. 1E) T cells, indicating the autocrine C3‐CD46 axis is perturbed in T cells from SLE patients.

### Dysregulation of Th1 contraction (IL‐10 switching) persists over time in SLE patients

**Figure 2:**
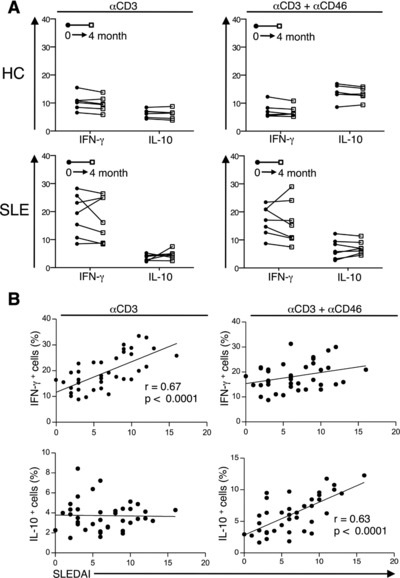
Dysregulation of Th1 contraction (IL‐10 switching) persists over time in SLE patients. (A) CD4^+^ T cells of healthy controls (HCs, *n* = 6) and SLE patients (*n* = 7) were activated for 36 h as indicated, analyzed by flow cytometry, and plotted as percentages of either total numbers of IFN‐γ‐positive (IFN‐γ^+^IL‐10^−^ and IFN‐γ^+^IL‐10^+^) or IL‐10‐positive (IFN‐γ^+^IL‐10^+^ and IFN‐γ^−^IL‐10^+^) cells. T cells were isolated again from the same HCs and SLE patients 4 months later and analyzed in the same fashion. (B) The relationship between disease activity index (SLEDAI) and total numbers of CD46‐induced IL‐10‐positive T cells is shown, with each plot displaying the total numbers of IFN‐γ‐positive (IFN‐γ^+^IL‐10^−^ and IFN‐γ^+^IL‐10^+^) or IL‐10‐positive (IFN‐γ^+^IL‐10^+^ and IFN‐γ^−^IL‐10^+^) cells. *p* and *r* values in (B) were calculated using the Pearson correlation coefficient.

SLE commonly exhibits fluctuation in activity over time and we therefore asked whether the CD46‐mediated switching of Th1 cells into an IL‐10‐producing regulatory and contracting phase was persistently defective in SLE. To this end, we first analyzed the number of total IFN‐γ (IFN‐γ^+^IL‐10^−^ andIFN‐γ^+^IL‐10^+^) or IL‐10‐positive (IFN‐γ^+^IL‐10^+^ and IFN‐γ^−^IL‐10^+^) T cells from HCs and individuals with SLE after CD3 and CD3+CD46 activation (Fig. 2A, time ‘0’). After 4 months, the same HCs and SLE patients provided a second blood sample and the assays were repeated (Fig. 2A, time ‘4 months’). These experiments demonstrated that in the majority of those with SLE, the reduction in effective IL‐10 switching and Th1 contraction was sustained over time and remained part of their pathological CD4^+^ T‐cell activation profile. We next assessed for a possible correlation between SLE disease activity, defined by the disease activity index (SLEDAI). We observed a significant positive correlation between increased numbers of IFN‐γ‐secreting (but not for IL‐10‐secreting) T cells after CD3 activation and disease activity (Fig. 2B). Somewhat surprisingly, the numbers of IL‐10‐secreting cells (after CD3+CD46 activation) also correlated positively with disease activity (Fig. 2B). Of note, the IL‐10‐secreting cells in this analysis contained both IFN‐γ^+^IL‐10^+^ and IFN‐γ^−^IL‐10^+^ cell populations and it was previously shown that the IFN‐γ^+^IL‐10^+^ T cell population in RA patients contributed mostly to disease 9.

### Th1 cells from SLE patients fail to supress T‐cell proliferation and to support B‐cell maturation

**Figure 3:**
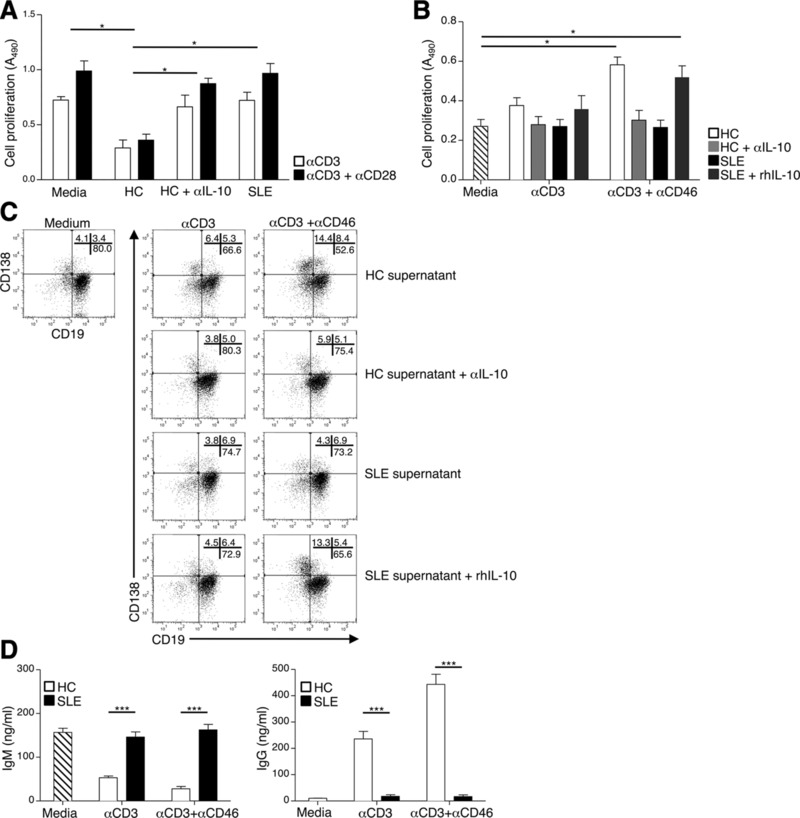
Th1 cells from SLE fail to control bystander T cell proliferation and to support B cell maturation. (A)T cells from healthy controls were activated with either anti‐CD3 or anti‐CD3 and anti‐CD46 in fresh media or supernatants derived from CD3^+^CD46‐activated T cells of HCs (with and without addition of a function neutralizing Ab to IL‐10) or in supernatants derived from CD3^+^CD46‐activated T cells from SLE patients. Cell/supernatant mixtures were activated for 6 days and proliferation analyzed. Data are shown as mean + SD and are representative of five experiments (*n* = 5) using cells or supernatants from a different HC and SLE patient each time. (B and C). B cells from HCs were cultured in media or mixed with supernatants of CD4^+^ T cells derived from CD3 or CD3+CD46‐activated T cells (36 h) from HCs or a patient with SLE. Cell supernatants from HCs were also supplemented with anti‐IL‐10 Ab while cell supernatants from SLE patients were supplemented with rhIL‐10. Cell/supernatant mixtures were cultured for 10 days and (B) B‐cell proliferation and (C) plasma blast cell differentiation (CS138^high^CD19^low^ B cells) measured. Data are shown as mean + SD or one representative FACS plot derived from five experiments (*n* = 5). (D) T cells from SLE patients fail to support B‐cell class switching. B cells from HCs were cultured for 10 days in supernatants from HC or SLE patient T cells activated with either anti‐CD3 or anti‐CD3 and anti‐CD46 and IgM and IgG secretion assessed by ELISA (*n* = 5). *p* values in (A) and (B) were calculated using the Mann–Whitney U test. *p < 0.05.

The inhibitory effect of IL‐10 co‐produced by contracting Th1 cells plays a key role in normal Th1 self‐regulation and regulation of bystander T cells and therefore maintenance of peripheral tolerance 22.Hence, normally, cell supernatants isolated from CD3+CD46‐activated T cells are suppressive toward bystander T cells due to their high IL‐10 content 9, 15. To assess the regulatory capacity of CD46‐activated SLE T cells, freshly isolated autologous CD4^+^ T cells were activated with antibodies to either CD3 or CD3+CD28 in the presence of supernatants derived from CD3+CD46‐activated T cells from HCs or SLE patients and cell proliferation measured 6 days post‐activation. The supernatants derived from control T cells showed the expected suppressive capacity and significantly inhibited proliferation of CD3+CD28‐activated T cells. This suppression was mediated by IL‐10 as neutralization of IL‐10 fully restored bystander T‐cell activation (Fig. 3A). In contrast, cell supernatants from CD3+CD46‐activated SLE T cells had no suppressive capacity and allowed for bystander proliferation comparable to HC T cells cultured in fresh media (Fig. 3A).

Normally, CD46‐activated CD4^+^ T cells support B‐cell proliferation and Ig class switching in an IL‐10‐ and CD40L‐dependent manner 23. We therefore investigated the effect of CD46‐activated supernatants from HCs and SLE patients on B‐cell maturation and class switching. B cells cultured in supernatants derived from CD3+CD46‐activated HC T cells showed significantly higher proliferation compared to B cells cultured in supernatants of SLE T cells or fresh media (Fig. 3B). The enhanced proliferation induced by HC T cell supernatants was IL‐10‐dependent as the addition of a neutralizing antibody to IL‐10 abolished the increase proliferation (Fig. 3B). Supplementation of patients’ samples with rhIL‐10 restored the support of B cell proliferation given normally by T cell supernatants (Fig. 3B). Similarly, supernatants from CD3+CD46‐activated HC T cells also supported plasmablast (CD19^low/−^/CD138^+^ cells) differentiation in an IL‐10‐dependent manner as well as induction of IgM to IgG class switching (Fig. 3C and D). In contrast, supernatants from SLE T cells failed to support plasmablast differentiation or immunoglobulin class switching 23.

### Increased shedding of sCD46 by SLE T cells correlates with lack of Th1 contraction

**Figure 4:**
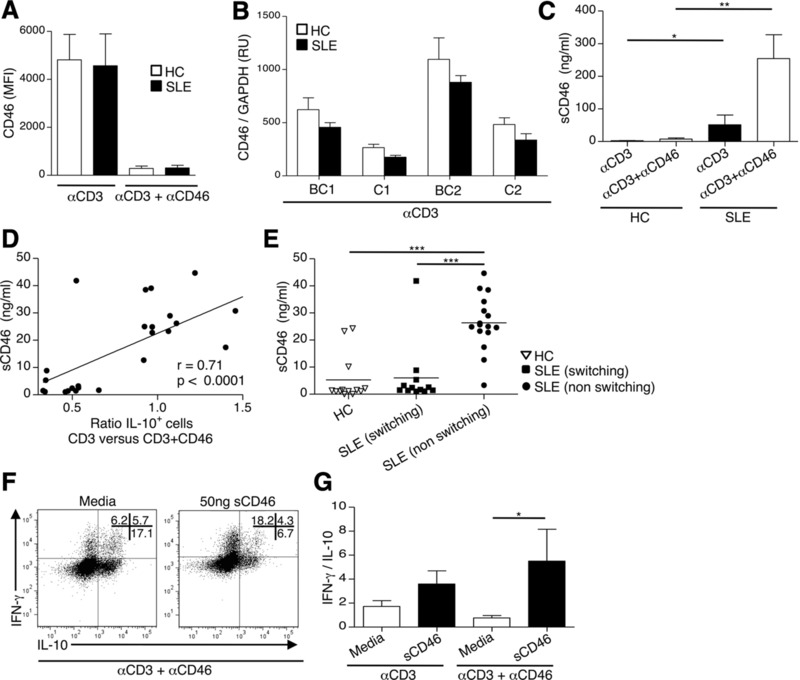
Increased CD46 shedding by T cells from SLE patients contributes to faulty IL‐10 switching. (A and B) T cells of healthy controls (HCs, *n* = 10) and SLE patients (*n* = 15) were activated as indicated (36 h) and (A) CD46 surface expression analyzed by FACS and (B) CD46 isoform expression assessed by RT‐PCR in CD3‐activated T cells. (C) Increased shedding of CD46 by CD3+CD46‐activated Th1 cells from SLE patients. T cells of HCs (*n* = 11) and SLE patients (*n* = 13) were stimulated for 36 h and sCD46 concentrations in cell supernatants analyzed by ELISA. (D) Correlation analysis of sCD46 and IFNγ^+^IL‐10^+^ Th1 cells from patients with SLE (*n* = 26) depicted as ratio of IL‐10^+^ cells after CD3+CD46 activation. Note, low values denote successful “switching” while high values correlate with “non‐switching”. (E) Increased sCD46 concentrations in sera of SLE patients with impaired switching (*n* = 16) compared to SLE patients with normal switching (*n* = 12) and HCs (*n* = 16) after CD3+CD46 activation. (F) T cells of HCs were stimulated for 36 h as indicated either with or without addition of sCD46 (50 ng/mL) and. IFN‐γ and IL‐10‐positive cells enumerated. Shown is a representative FACS plot of five similarly performed experiments. (G) sCD46 tips the balance toward IFN‐γ secretion. T cells were activated as shown with or without addition of sCD46 and the ratio of IFN‐γ to L‐10 in supernatants assessed at 36 h. *p* and *r* values in (D) were calculated using the Pearson correlation coefficient. *p* values in (C), (E) and (G) were calculated using the Mann–Whitney U test. *p < 0.05; **p < 0.005, ***p < 0.0001.

We next investigated the underlying cause of reduced IL‐10 production by SLE CD4^+^ T cells. To start, the levels of cell‐surface CD46 were measured together with CD46 transcript isoform expression 24 in resting and activated T cells. Resting and CD3‐activated T cells isolated from HC and SLE patients showed comparable levels of CD46 expression following CD3‐activation (Fig. 4A) and also no differences in the CD46 isoform (BC1, C1, BC2, and C2) expression pattern (Fig. 4B). Further, CD46 surface levels were reduced to the expected and equal amounts upon CD3+CD46 stimulation on HC and SLE T cells (Fig. 4A). Thus, initial CD46 expression levels, and their isoform expression pattern and the ability to induce CD46 shedding on resting and CD3‐activated T cells were comparable between HC and SLE patients.

Because increased levels of soluble CD46 (sCD46) have been observed in SLE sera^21^ and we have demonstrated that sCD46 abrogates the CD46‐mediated co‐induction of IL‐10 in Th1 by interfering with normal Notch signaling 10, we next assessed whether CD46 shedding may be altered in SLE T cells. Indeed, while supernatants from CD3 and CD3+CD46‐activated (after 36 h) HC T cells contained ∼2 and ∼8 ng/mL sCD46, respectively, SLE T cells ‘produced’ ∼50 ng/mL sCD46 with CD3 activation and up to ∼300 ng/mL after CD3+CD46 stimulation (Fig. 4C). The relationship between sCD46 and defective Th1 contraction was quantitative: increased sCD46 levels correlated positively with the increase of ‘faulty/non‐suppressive’ IL‐10‐positive cells in the SLE patients (Fig. 4D). Furthermore, sCD46 was mainly detectable in samples from SLE patients with impaired IL‐10 switching, but not in those that had no switching defect (Fig. 4E). Importantly, the addition of 50 ng/mL of rsCD46 to control T cells during CD3+CD46 activation was sufficient to inhibit normal IFN‐γ to IL‐10 switching (Fig. 4F) and to tip the balance significantly toward IFN‐γ secretion as assessed by the IFN‐γ:IL‐10 producing T cells (Fig. 4G). Thus, T cells from patients with SLE have increased CD46 shedding upon activation and such (local) increased amounts of sCD46 prevent normal Th1 contraction, at least in vitro.

### MMP‐9 mediates CD46 shedding by activated human CD4^+^ T cells

**Figure 5:**
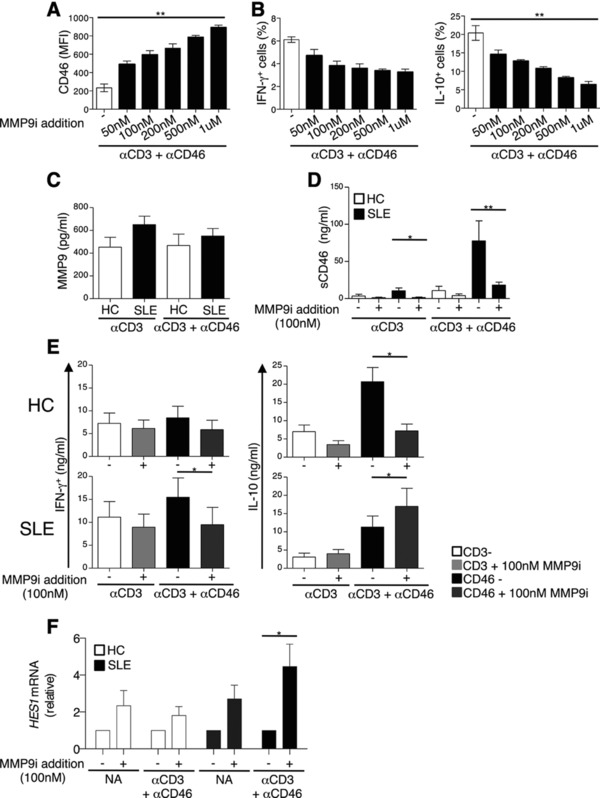
MMP‐9 inhibition restores Th1 contraction in SLE patients. (A and B) T cells were CD3^+^CD46 activated for 36 h in the presence of increasing concentrations of an MMP‐9‐specific inhibitor and (A) CD46 expression assessed by FACS and (B) IL‐10 and IFN‐γ secretion measured by CBA. The mean + SD of the results of three independent experiments on three healthy controls is shown. (C). T cells of HCs (*n* = 6) and SLE patients (*n* = 10) were activated for 36 h as indicated and MMP‐9 secretion analyzed by ELISA. (D) T cells of HCs (*n* = 8) and SLE patients (*n* = 10) were activated as indicated for 36 h and sCD46 release measured. (E) T cells of HCs (*n* = 10) and SLE patients (*n* = 10) were activated as indicated for 36 h with or without the addition of the MMP‐9 inhibitor and IL‐10 and IFN‐y secretion measured. (F) MMP‐9 inhibition restores normal Notch1 activity in T cells from SLE patients. Quantitative RT‐PCR analysis of *HES1* gene transcription in resting or CD3+CD46‐activated T cells (36 h) from HCs and SLE patients with or without addition of MMP‐9 inhibitor. mRNA levels were normalized to S18 ribosomal mRNA and depicted relative to non‐activated cells. Data are derived from two independent experiments with conditions performed in triplicate using T cells from four HCs and five SLE patients. *p* values in (D) and (E) were calculated using the paired *t* test, *p < 0.05; **p < 0.005.

Previous studies have shown that MMPs and their closely related family of ADAMs can process the ectodomain of CD46 upon its activation via crosslinking in different cell types 18, 25, 26. We hypothesized that MMP‐9 (gelatinase B) was a strong candidate in CD46 ectoderm shedding; first, because microarray analysis of CD3+CD46‐activated CD4^+^ T cells revealed elevated levels of *MMP9* transcripts 11, and second, because anti‐MMP‐9 autoantibodies have been reported in SLE 27. Indeed, blockade of MMP‐9 *via* a specific MMP‐9 inhibitor during T cell activation led to retention of CD46 on the cells surface in a dose‐dependent manner (Fig. 5A). The MMP‐9 inhibition‐mediated decrease in CD46 shedding led to a modest reduction in IFN‐γ secretion but reduced IL‐10 switching significantly (Fig. 5B). Of note, although elevated levels of MMP‐9 have been reported in sera of patients with SLE 20 we did not observe a difference in MMP‐9 released by activated control or SLE T cells (Fig. 5C). Nonetheless, addition of the specific MMP‐9 inhibitor to activated HC T cells not only reduced sCD46 generation but fully normalized sCD46 generation by SLE patients’ T cells (Fig. 5D). Importantly, inhibition of MMP‐9 activity also restored the impaired CD46‐mediated IL‐10 switching and contraction of Th1 cells observed in SLE by increasing IL‐10 production significantly and tipping the IFN‐γ to IL‐10 level ratio back towards IL‐10 (Fig. 5E). MMP‐9 inhibition reduced IFN‐γ production modestly in HC T cells but significantly prevented IL‐10 switching (Fig. 5E). The MMP‐9 inhibitor has different effects with regard to the IFN‐γ to IL‐10 production and/or ratio in HC and SLE T cells because MMP‐9 inhibition in HC T cells diminishes CD46 surface shedding and the release of the CD46 ‘brake’ on Notch signaling (and hence reduces both initial IFN‐γ induction and subsequent IL‐10 switching) while MMP‐9 inhibition in SLE T cells reduces the abnormally high generation of sCD46 to levels now allowing for normal IL‐10 switching. CD46‐driven IL‐10 induction in T cells is accompanied by Notch‐induced induction of *HES1* and increased levels of sCD46 in T‐cell cultures of HC interferes with *HES1* induction 10. In line with this observation, restoration of IL‐10 secretion in SLE T cells with MMP‐9 inhibition is indeed accompanied by a significant increase in *HES1* mRNA transcription (Fig. 5F), an effect not present in HC T cells. Thus, these data are in line with our published studies demonstrating that coordinated CD46 surface processing during and after Th1 induction is needed for normal Th1 induction and contraction 10 and support the conclusion that MMP‐9 is a physiological proteinase in such CD46 processing on CD4^+^ T cells.

## Discussion

Many abnormalities in B‐ and T‐cell compartments have been reported in SLE and these include, uncontrolled antibody production against self‐antigens, hyperactive secretion of pro‐inflammatory cytokines by effector T cells and possibly defects in natural regulatory T‐cell numbers and/or function (Tregs) 1. We here report for the first time a defect in the CD46‐mediated IL‐10 co‐expression and hence normal contraction of Th1 cells in the CD4^+^ T‐cell pool of patients with SLE. Mechanistically, this is driven by pathologically increased cleavage of CD46 from the T‐cell surface through the matrix metalloprotease MMP‐9 leading to subsequent dysregulation of Notch‐mediated signals that prevent normal Th1 shut down (Supporting Information Fig. 1).

This finding aligns well with the recent understanding that signals mediated by the Notch system 28 and by activation of fragments from intracellularly processed complement components C3 and C5 in human CD4^+^ T cells are critical to normal Th1 induction and contraction 29, 30, 31. Particularly the TCR‐induced autocrine engagement of the C3aR and of CD46 (via C3a and C3b, respectively) is a prerequisite for normal IFN‐γ secretion and Th1 induction in human CD4^+^ T cells. Furthermore, an as yet undefined signaling crosstalk between CD46 and the IL‐2R (after successful Th1 induction) contributes to IL‐10 co‐expression in Th1 cells and their transition into a suppressive and (self)regulating contraction phase 9. Similarly, coordinated Notch signaling via engagement with Notch ligands such as Jagged1 and 2 and/or Delta1‐4 is required for normal Th1 biology 19, 32 – hence, dysregulations in Notch and/or CD46‐mediated T‐cell signals contribute to autoimmune diseases such as RA and MS 9, 16.

Although it is now also increasingly appreciated that that the exact temporal and spatial control of such complex Notch and complement receptor/regulator crosstalk is key to normal Th1 induction and contraction, our understanding of how this is achieved is still limited. Both CD46 and Notch are cleaved via ADAMs from the cell surface upon their engagement by their respective ligands and elevated levels of sCD46 in sera of patients with active SLE, RA, Sjogen's syndrome, and MS have been observed 20, 21. Uncontrolled activation of the complement system has previously been linked with SLE leading to hypocomplementemia and complement deposition at sites of tissue injury 33, however, these effects were traditionally thought to be dependent on liver‐derived and serum‐active complement. Although one study connected mutations in CD46 with increased risk for onset of lupus nephritis 34 no work has so far been performed on the role for the sCD46 shed by (immune) cells in autoimmune disease.

Cell surface expression of CD46 is normally tightly regulated by MMPs and their closely related family of ADAMs 18, 26. While the biological significance of CD46 shedding remains unresolved for most cells, we recently demonstrated that soluble CD46 (sCD46) modulates Notch1‐mediated signals on T cells and impacts significantly on IFN‐γ to IL‐10 switching and Th1 contraction 10. Thus, our novel observation here that increased MMP‐9‐mediated CD46 shedding by CD4^+^ T cells from patients with SLE contributes to their defective IL‐10 switching and Th1 contraction may deliver for the first time a mechanistic explanation for the correlation between increased sCD46 and autoimmune disease. Hypomethylation of the *MMP9* promoter 35 and unrestrained MMP‐9 protein activity has indeed been reported in SLE and further supports our notion that this protease is important in Th1 biology via regulation of CD46 surface levels 27, 36.

The underlying reason for the increased MMP‐9 expression in T cells from patients with SLE – and whether this activity is a direct cause or a consequence of perturbed upstream signals – is currently unclear. Although IL‐17 can induce increased MMP‐9 expression in cells 37, we did not observe an increase in IL‐17 production by T cells from SLE patients under any activation conditions tested (Supporting Information Fig. 2). TCR activation in conjunction with CD46 co‐stimulation increases MMP‐9 expression 11 and we observed significantly increased generation of the CD46 ligand C3b in the patients’ T cells (Fig. 1E). Since autocrine CD46 activation also increases T‐cell intrinsic C3 expression via a positive feedback loop 11, one possibility is that dysregulated autocrine CD46 signaling – via increased intrinsic C3 activation – itself contributes to increased MMP‐9 expression. This notion aligns with our finding that CD46‐driven IL‐2R assembly (Supporting Information Fig. 1) and Notch up‐regulation (data not shown) are perturbed in the patients’ T cells. Notably, failure of Notch up‐regulation in activated T cells from SLE patients has been connected with an increase in disease activity 38. Interestingly, the CD46‐triggered nuclear translocation of *IL2* transcriptional regulators ICER and CREM (necessary for the switch of Th1 cells into an IL‐10‐producing regulatory phenotype) 9 remains unaffected (data not shown) suggesting that only certain aspects of CD46 signals are altered in SLE T cells.

In line with our previous studies demonstrating that reduced IL‐10‐switching in T cells from patients with RA correlates with the lack of suppressive capacity, T cells from SLE patients also fail to control bystander T‐cell activation in an IL‐10‐dependent manner (Fig. 3A). This observation may partly explain the hyperactive Th1 response in SLE 39. Furthermore, we have previously shown that IL‐10 co‐producing Th1 cells are ‘prime’ activators of B cells and strongly support class switching in an IL‐10‐dependent fashion 23. T‐cell supernatants from SLE patients mediated reduced plasmablast induction and class switching and this ‘defect’ could be fully rescued by addition of rhIL‐10 to cultures (Fig. 3C and D). This finding suggests that the increased amounts of sCD46 present in the T‐cell supernatants of SLE patients had no impact on the B‐cell activation parameters measured in our system and could indicate that the ‘action’ of sCD46 is confined or targeted to specific cell populations.

Although we have shown here that increased sCD46 affect normal Notch‐activity and that these signals can be ‘normalized’ via MMP‐9 inhibition, the elevated sCD46 generated by SLE T cells will likely have additional effects that we have not yet accounted for. For example, sCD46 binds C3b and may therefore compete with other C3b/iC3b‐fragment binding receptors for this ligand and modulate their contributions to immune cell effector functions (Supporting Information Fig. 2); particularly in vivo where T cells operate in the context of other immune cells and tissues and not in isolation as assessed in the in vitro system applied here. The idea that much more work is required to understand the complex in vivo role of autocrine complement in health and disease is underpinned by the finding that, unexpectedly, increased numbers of IL‐10‐producing (and not IFN‐γ producing) T cells after CD3+CD46 activation correlate with the highest SLEDAI score in SLE patients (Fig. 2B). The explanation for this observation is not clear but could reflect an increased number of activated and/or memory‐like T‐cell generation in the SLE patients (as previously observed, 40) as we and others have shown that repeated T‐cell stimulation correlates with accelerated IL‐10 switching and memory induction 9, 41. In this regard, although CD46 is now firmly recognized as a key modulator of human Th1 biology, fully dissecting its diverse in vivo functions on a range of cell populations remains a challenge as mice do not express CD46 on somatic cells and tissues and a homologue that fully recapitulates CD46's functions has so far not been identified in mice 29.

In summary, we demonstrate here that MMP‐9‐mediated shedding of CD46 is an integral part of autocrine Th1 regulation and that this pathway is dysregulated in T cells from patients with SLE. Thus, targeting MMP‐9 activity could potentially be a promising candidate in the fight against SLE and possibly other autoimmune diseases.

## Methods

### Healthy donors and patients

A total of 45 patients with SLE fulfilling the revised American College of Rheumatology classification criteria were recruited from the Hammersmith and St. Thomas` Hospital, London. Disease severity was assessed using the SLE disease activity index (SLEDAI). The mean age of patients was 37 years, (M:F = 0.21). Thirty‐eight healthy volunteers had a mean age 34 years, (M:F = 0.35). All the patients were treated with hydroxychloroquine, some were also on low dose prednisolone and/or mycophenolate. No patients were on high dose corticosteroids or had ever received anti‐CD20 therapy. Informed consent was obtained and ethically approved: REC 12/LO/1273 and 07/H0718/49.

### Antibodies, recombinant proteins, and inhibitors

Cell stimulating antibodies to CD3 (clone OKT‐3), CD28 (clone AF‐342‐PB) and CD46 (clone 344519) were all purchased from R&D Systems. Neutralizing antibodies to IL‐4 (clone 8D4‐8) and IL‐10 (clone JES3‐19F1) were both from BD Pharmingen. Antibodies used in flow cytometric analysis were FITC‐labeled anti‐CD46 (clone MEM‐258), BV421‐labeled anti‐CD122 (clone TU27), PE‐labeled anti‐CD132 (clone AG184) from BioLegend, and FITC‐labeled anti‐CD19 (clone 4G7‐2E3) and APC‐labeled anti‐CD138 (clone 359103), both from R&D Systems, while the APC‐labelled anti‐CD25 (clone M‐A251) was purchased from BD Pharmingen. Recombinant human IL‐2 and IL‐10 were purchased from PeproTech and soluble CD46 was prepared as previously described.^10^ The MMP‐9 inhibitor was purchased from Calbiochem (CAS 1177749‐58‐4) and used at the concentrations indicated in the respective experiments.

### PBMC, T‐cell and B‐cell isolation

Peripheral mononuclear cells (PBMCs) were isolated from anticoagulated venous blood using Ficoll density centrifugation (Sigma‐Aldrich Histopaque‐1077). CD4^+^ T cells were isolated utilizing CD4^+^ MicroBeads (Miltenyi Biotec), their purity was >97% in all experiments. CD19^+^ B cells were isolated using Dynabeads (Invitrogen), their purity was >95% in all experiments.

### B cell and T cell in vitro activation

Purified CD19^+^ lymphocytes (1 × 10^5^ cells/well) were cultured in 96‐well plates in 50 μL medium (RPMI, 5%FBS) supplemented with 50 ng/mL rhIL‐10 (Miltenyi Biotec), 20 U rhIL‐2 (PeproTech), 0.1 μg/mL CD40L (Enzo Life Science) and 25 μg/mL anti‐human IgM F(ab’)_2_ (Jackson ImmunoResearch). Purified CD4^+^ lymphocytes (2.5 × 10^5^‐3.5 × 10^5^ cells/well) were cultured for 36 h with rhIL‐2 (PeproTech, 50U/mL) at 37°C, 5% CO_2_ in 48‐well plates that had been coated overnight with anti‐CD3 (2 μg/mL) (BioLegend, clone OKT3) +/‐anti‐CD46 (2 μg/mL) (R&D Systems, clone 344519). To assess B‐cell activation, 100 μL of T‐cell supernatant was added to B cells and cultured for 5–10 days at 37°C, 5%CO_2_. To block the activity of IL‐10, a neutralizing mAb to IL‐10 (2.5 μg/mL) was used. To restore B‐cell proliferation in mixtures containing T‐cell supernatants rhIL‐10 (1 μg/mL) was added.

### Suppression and proliferation assays

Purified CD4^+^ T cells (3 × 10^5^) were stimulated for 36 h on 48‐well plates with immobilized anti‐CD3, anti‐CD46, and rhIL‐2 (50 U/mL). Supernatants from these cells were harvested and transferred to freshly isolated CD4^+^ T cells. T cells cultured in medium served as a control. T cells/supernatant mixtures were activated for 6 days with anti‐CD3 ±anti‐CD28. To block the activity of IL‐10 in culture a neutralizing mAb to IL‐10 was used at a concentration of 2.5 μg/mL. Cell proliferation was measured using the CellTiter96 AQ_ueous_ One solution Cell Proliferation kit (Promega).

### Cytokine measurements

Cytokines were measured using the human IFN‐γ or IL‐10 Cytokine Secretion Assay (Miltenyi Biotec) in combination or the T_H_1/T_H_2 Cytometric Bead Array (BD Bioscience). Samples were analyzed by Flow cytometry on a BD FACS Canto machine and data analyzed using FlowJo software (V10.0.6).

### Measurements of soluble CD46, MMP‐9, C3b, and Ig in cell supernatants by ELISA

Amounts of sCD46 in culture supernatants was measured by sandwich ELISA (detection range: 7.8–500 ng/mL): anti‐CD46 (clone GB‐24),^38^ and biotin anti‐CD46 (clone MEM‐258, Novus Biologicals). IgM and IgG in B cell tissue culture supernatants were analyzed using the human IgM (detection range: 15.6–1000 ng/mL) and IgG (detection range 7.8–1000 ng/mL) ELISA quantification (Bethyl Laboratories). MMP‐9 in T cell tissue culture supernatants was measured using the LegendMax^TM^ ELISA (BioLegend; detection range: 62.5–4000 pg/mL). C3b was analyzed using the Complement C3b human ELISA (Abcam; detection range: 0.15–10 ng/mL).

### RT‐PCR for CD46 isoforms and quantitative RT‐PCR for HES1 mRNA

Total RNA was extracted from CD4^+^ T cells using the RNeasy mini kit (Qiagen). To analyze the expression pattern of CD46 isoforms the following CD46‐specific primers were used:5’GTGGTCAAATGTCGATTTCCAGTAGTCG‐3’ (forward) 5’CAAGCCACATTGCAATATTAGCTAAGCCACA‐3’ (reverse). A total of 10 ng of total RNA was used and RT‐PCR was performed with the One‐Step RT‐PCR (Qiagen). PCR products were analyzed using the GelAnalyzer2010 (Agilent Technologies). For *HES1* expression the following primers were used: 5′‐GAAGCACCTCCGGAACCT‐3′ (forward) 5′‐GTCACCTCGTTCATGCACTC‐3′ (reverse).

### Statistical analyses

Statistical analysis was performed using, where appropriate, either the two‐tailed Mann–Whitney U‐test, the Student's paired *t*‐test or the Pearson correlation coefficient.

## Author contributions

U.E. designed and performed experiments and wrote the manuscript. A.C. performed experiments, helped with the statistical analysis and discussed the data. C.L.P. performed experiments, G.L.F. performed experiments and discussed the data. C.K. designed experiments, discussed the data and wrote the manuscript and T.J.V. discussed the data and wrote the manuscript.

## Conflict of interest

The authors declare no financial or commercial conflict of interest.

**Abbreviation**
SLE: systemic lupus erythematosus

## Supporting information

Supporting Information Figure 1. Model suggestion about potential contribution(s) of dysregulated CD46 shedding during defective Th1 contraction in SLE.
Supporting Information Figure 2. IL‐17 production is not increased in CD4+ T cells isolated from patients with SLE.
Supporting Information Figure 3. Disturbed IL‐2 receptor assembly in CD3 and CD46‐activated CD4+ T cells of patients with SLE

Peer review correspondence
