# Supplementary material for: Dysregulated CD46 shedding interferes with Th1‐contraction in systemic lupus erythematosus
Source: Eur J Immunol. 2017 May 22;47(7):1200–10. doi: 10.1002/eji.201646822 (PMC5507296; doi:10.1002/eji.201646822)
Supplement: Supplementary file 1 — Supporting Information Figure 1. Model suggestion about potential contribution(s) of dysregulated CD46 shedding during defective Th1 contraction in SLE. Supporting Information Figure 2. IL‐17 production is not increased in CD4+ T cells isolated from patients with SLE. Supporting Information Figure 3. Disturbed IL‐2 receptor assembly in CD3 and CD46‐activated CD4+ T cells of patients with SLE [file EJI-47-1200-s001.pdf]

European Journal of Immunology

Supporting Information

for

DOI 10.1002/eji.201646822

Ursula Ellinghaus, Andrea Cortini, Christopher L. Pinder, Gaelle Le Friec, Claudia Kemper and Timothy J. Vyse

Dysregulated CD46 shedding interferes with Th1-contraction in systemic lupus erythematosus

© 2017 The Authors. *European Journal of Immunology* published by WILEY-VCH Verlag GmbH & Co. KGaA, Weinheim.

Supplementary figure 1

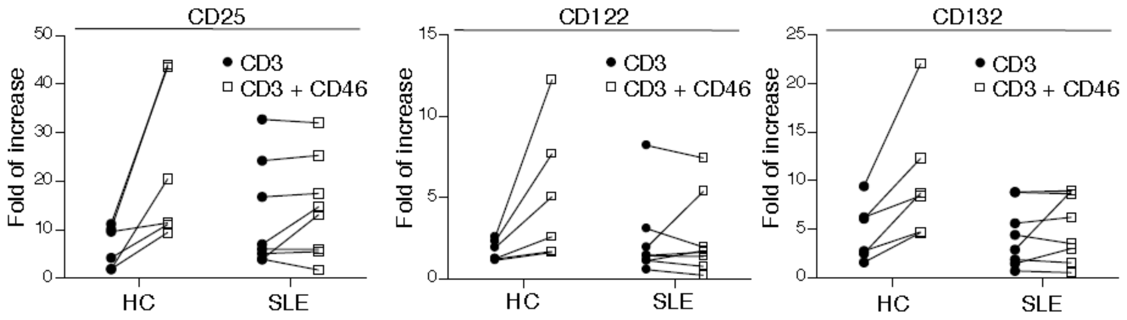

Supplementary Figure 1. Disturbed IL-2 receptor assembly in CD3 and CD46-activated CD4$^+$ T cells of patients with SLE. T cells of healthy controls (HCs, n=6) and patients with SLE (n=8) were activated as indicated and expression of the α-(CD25), β-(CD122) and γ-(CD132)-chain of the IL-2R analysed by flow cytometry. Data are plotted as the fold of increase of the IL-2R expression on CD3- and CD3 and CD46-activated CD4$^+$ T cells compared to expression levels on non-stimulated cells.

Supplementary Figure 2

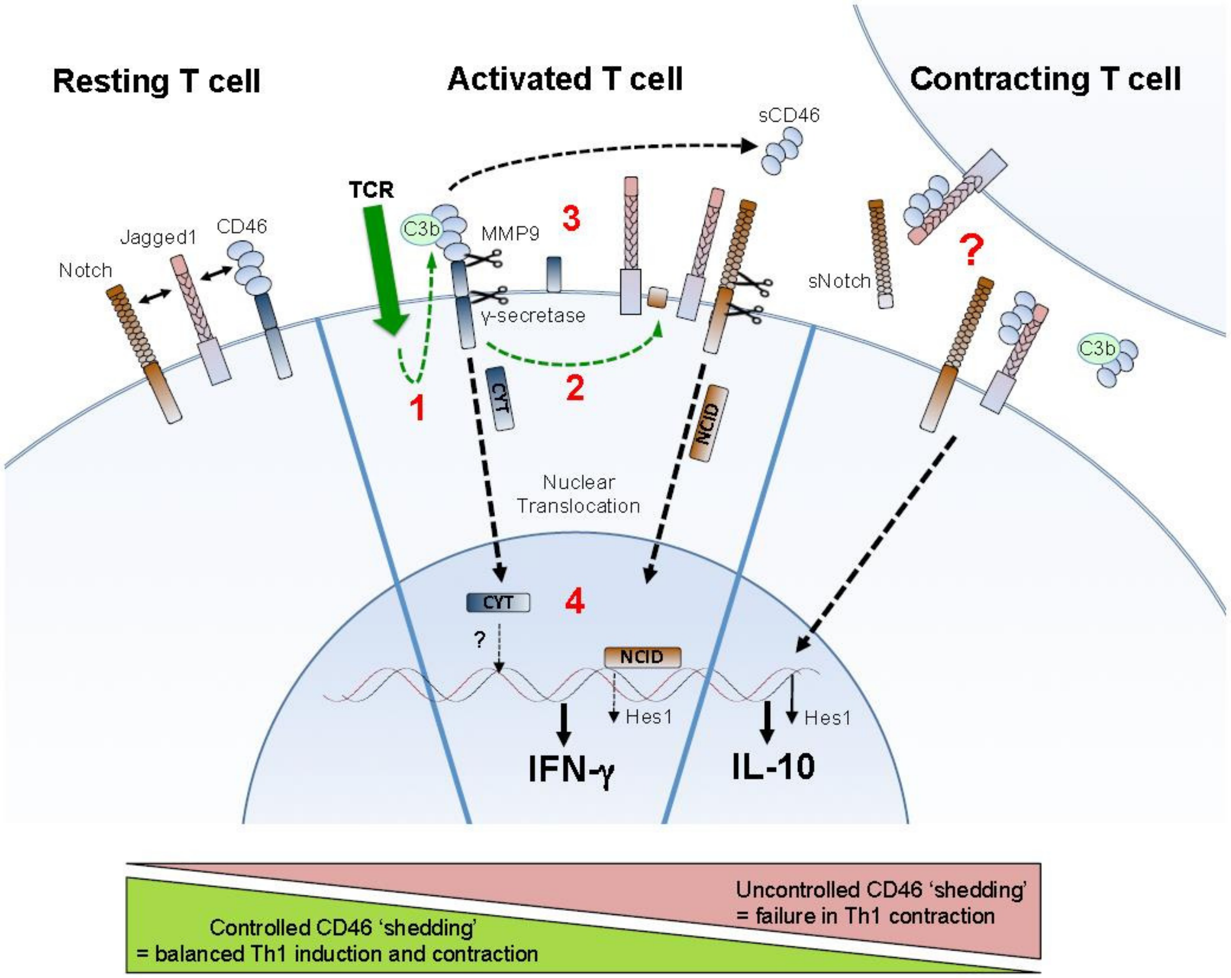

Supplementary Figure 2. Model suggestion about potential contribution(s) of dysregulated CD46 shedding during defective Th1 contraction in SLE. In the resting state, CD46, expressed on the T cell surface, tethers Jagged1 away from Notch1 and prevents a T cell-stimulating Jagged1-Notch interaction. Upon T cell receptor (TCR) stimulation, autocrine generated C3b engages CD46 (1) and CD46-mediated signals induce the up-regulation of Jagged1 and Notch (2) via yet undefined signalling pathways. TCR activation also induces processing of CD46 on the cell surface via metalloproteinase 9 (MMP9) (3) and intracellularly via γ-secretase (similar to Notch and Jagged1). Processed intracellular domains of Notch (NCID) and CD46 (CYT) translocate to the nucleus (4) where NCID regulates target gene transcription (such as Hes Family BHLH Transcription Factor 1, *HES1*). Nuclear translocation of CYT-1 of CD46 is a requirement for Th1 induction, however, the exact functional role of CD46's tails in the nucleus remain to be established. Similarly, although soluble CD46 (sCD46) generated via MMP9-mediated shedding contributes to normal IL-10 switching and Th1 contraction via fine-tuning Notch signalling, it is not yet clear whether this occurs via the regulation of *trans*- and/or *cis*-interactions between Notch and Jagged1. In addition, regulation of local amounts of C3b by sCD46 (competition with other C3b receptors) or other yet to be defined mechanism (denoted by a question mark) should also be explored.
