# Supplementary material for: Dysregulated CD46 shedding interferes with Th1‐contraction in systemic lupus erythematosus
Source: Eur J Immunol. 2017 May 22;47(7):1200–10. doi: 10.1002/eji.201646822 (PMC5507296; doi:10.1002/eji.201646822)
Supplement: Supplementary file 2 — Peer review correspondence [file EJI-47-1200-s002.pdf]

European Journal of Immunology

Dysregulated CD46 shedding interferes with Th1-contraction in systemic lupus erythematosus

Ursula Ellinghaus, Andrea Cortini, Christopher L. Pinder, Gaelle Le Friec, Claudia Kemper and Timothy J. Vyse

Correspondence: Prof. Timothy Vyse, Division of Genetics and Molecular Medicine, Department of Medical and Molecular Genetics, King's College London, Guy's Hospital, Great Maze Pond, London, United Kingdom

---

Review Timeline:	Submission date:	21-Nov-2016
	First Editorial decision:	23-Dec-2018
	Revision received:	01-Mar-2017
	Second Editorial decision:	24-Mar-2017
	Accepted:	20-Apr-2017

Handling Executive Committee member: Prof. Francesco Annunziato

Please note that the correspondence below does not include the standard editorial instructions regarding preparation and submission of revised manuscripts, only the scientific revisions requested and addressed.

First Editorial Decision - 23-Dec-2016

Dear Prof. Vyse,

Manuscript ID eji.201646822 entitled "Dysregulated CD46 shedding interferes with Th1-contraction in systemic lupus erythematosus" which you submitted to the European Journal of Immunology has been reviewed. The comments of the referees are included at the bottom of this letter.

A revised version of your manuscript that takes into account the comments of the referees will be reconsidered for publication. Should you disagree with any of the referees concerns, you should address this in your point-by-point response and provide solid scientific reasons for why you will not make the requested changes.

You should also pay close attention to the editorial comments included below. **In particular, please edit your figure legends to follow Journal standards as outlined in the editorial comments. Failure to do this will result in delays in the re-review process.**

European Journal of Immunology

Please note that submitting a revision of your manuscript does not guarantee eventual acceptance, and that your revision will be re-reviewed by the referees before a decision is rendered.

If the revision of the paper is expected to take more than three months, please inform the editorial office. Revisions taking longer than six months may be assessed by new referee(s) to ensure the relevance and timeliness of the data.

Once again, thank you for submitting your manuscript to European Journal of Immunology and we look forward to receiving your revision.

Yours sincerely,
Laura Soto Vazquez

On behalf of Prof. Francesco Annunziato

Editorial Office
European Journal of Immunology
e-mail: ejied@wiley.com
www.eji-journal.eu

****

Reviewer: 1

Comments to the Author
The work by Ellinghaus Ursula and coauthors investigates a field of potential clinic interest and in general is well planned, but some data seem to contradict the general message of the work, thus there are some points that need to be satisfied.

Major points:
1. In figure 2 B it is shown that IFN-gamma positively correlate with SLEDAI when T cell are stimulated by anti-CD3 mAbs, while IL-10 did not, as expected. But when cells are stimulated by antCD3 + anti-CD46 the data are just the opposite. How the authors explain this? The assessment of both IL-10 and IFN-gamma on sera of SLE patients, and their correlation with SLEDAI, is needed to understand what of the two phenomena happens in vivo.
2. CD46 MFI is strongly reduced in both HC and SLE patients when cells are stimulated by anti-CD3

+ anti-CD46 (figure 4A). This data is very likely an artifact due to CD46 ligation by the stimulating anti-CD46 antibody used in cell culture. mRNA evaluation of CD46 should clarify this point

3. The authors show a direct correlation between IL-10 producing cells and the amounts of sCD46 (figure 4 D), this result is in contrast with what expected and with the other data of the work, (i.e. figure 4 E and F). Since SLE patients are composed by IL-10 switching• and not switching• subjects (fig 4E), could the correlation be affected by a non-homogeneous group of patients?
4. IL-17 strongly induces MMP9, did the author evaluated the production of IL-17 by T cells from SLE patients?

Minor points:

1. P values are not reported in figure 3 D
2. In figure 3 legend the last sentence starts with (C)• instead of (D)•
3. MMP9 inhibitor has opposite effects with regard to IFN-gamma production in HC and in SLE patients (figure 5B and 5E). The author should speculate on some possible explanation.
4. In figure 5B a p value is reported, while in the text it results that the differences are not significant

Reviewer: 2

Comments to the Author

The present manuscript by Ellinghaus et al. entitled Dysregulated CD46 sheddig interferes with Th1-contraction in systemic lupus erythemtosus investigated the pathological role of CD46 and its regulatory pathway in patients with SLE. The authors hypothesized that the CD46 pathway plays a pivotal role in the activation of the Th1 response. Therefore the authors included 45 SLE patients and 38 healthy volunteers. First IFN-Î³ and IL-10 secretion in isloated CD4+ cells was performed with anti-CD3 and anti-CD46 stimulation resulting in predominantely Th1 response. This pattern seems to be stable over a period of several weeks demonstrated in a rather small subgroup of patients analysed at two visits. The decreased amount of IL-10 production leads to reduced capacity to inhibit B- and T-cells proliferation which has been elegantly assed in two different proliferation tests.

To test whether shedding of sCD46 is the underlying mechanisms of the Th1/IL-10 imbalance the authors studied CD46 expression in resting and activated cells and sCD46 in supernatants. The author concluded that increased CD46 shedding upon activation and increased sCD46 prevent Th1 contraction in vitro. Last the authors showed an effect of MMP-9 inhibition which might indicate that MMP-9 is a crucial factor in mediating CD46 shedding.

The present manuscript deals with an important topic and the data are sound. The introduction is well written. The patients and methods seem to be adequate. Some minor comments should be addressed.

1. SLEDAI was used as stated in the results. This should be mentioned in the method part. Were the patient on immunosuppressive drugs? Which were used? 2. The detection levels oft he ELISA should be mentioned in the method part. 3. Figure 1B and 2A have Total cyt+ cell (%) on the y-axis. What does cyt+ mean? Should be clarified. 4. Perhaps I do understand it not correct but to my knowledge Figure 4 A shows CD46 expression after stimulation with CD3 and after stimulation with anti-CD3 and anti-CD46. Tom e ist not surprising that anti-CD46 stimulation decreases CD46 expression because the receptor is blocked? Are you sure that you do not detect sCD46 which might be partially the anti-CD46 you added as stimuli? (Figure 4C)

Beside the above mentioned comments the present manuscript addresses an clincal interesting topic. The presentation of the data is clear. The supplementary figure 2 is very helpful.

First Revision – authors' response - 01-Mar-2017

Naturally, we acknowledge the Referees' commitment of time and effort and particularly welcome their many positive comments. Below we respond to individual points.

REVIEWER 1:
The work by Ellinghaus Ursula and coauthors investigates a field of potential clinic interest and in general is well planned, but some data seem to contradict the general message of the work, thus there are some points that need to be satisfied.

Major points:
1. In figure 2B it is shown that IFN-gamma positively correlate with SLEDAI when T cell are stimulated by anti-CD3 mAbs, while IL-10 did not, as expected. But when cells are stimulated by antCD3 + anti-CD46 the data are just the opposite. How the authors explain this? The assessment of both IL-10 and IFN-gamma on sera of SLE patients, and their correlation with SLEDAI, is needed to understand what of the two phenomena happens in vivo.
This is indeed a key point made by the reviewer and we have below (and in the revised version of the manuscript) now included a potential explanation of this rather counterintuitive observation.
A positive correlation between the SLEDAI and serum IL-10 levels has been observed previously also by other groups in the field (Godsell et al., Scientific Rep, 2016; Yang et al., J Biomed Res, 2015). Importantly, IL-10 is not only produced by 'switching' Th1 cells but also by monocytes (and possibly neutrophils), thus, it is likely not possible to unequivocally attribute the levels of IL-10 in serum to either one of these cells types.
To exclude that the patients with the highest SLEDAI scores depicted in Figure 2B may behave differently

we have performed cytokine analyses of their T cells and confirmed that their T cells still produce less IL-10 in proportion to IFN-γ when compared to HC T cells (Figure 1, below) (similar to what we observed for T cells from patients with rheumatoid arthritis (Cardone et al., Nat Immunol, 2010).

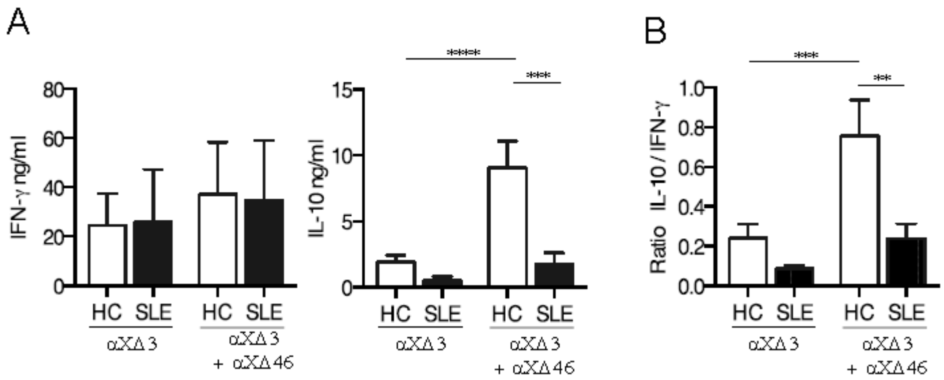

Figure 1. IFN-γ and IL-10 levels in cell supernatants of CD3- and CD3+CD46-activated T cells from healthy controls (HCs, white bars) and SLE patients (black bars) 36h post activation. (A) Concentration of IL-10 and IFN-γ in ng/ml. (B) Ratio of the concentrations of IL-10 over IFN-γ.

We would like to suggest the following hypothesis to explain the unexpected correlation of high % of IL-10-positive cells in our study with high(est) SLEDAI score: Others and we have shown that naïve CD4+ T cells produce little IFN-γ and no IL-10 when initially stimulated (Cardone et al., Nat Immunol, 2010; Meiler et al., JEM, 2008). Only upon restimulation, T cells start to produce IFN-γ and, upon each further round of restimulation then "switch" faster and faster into a regulatory IL-10-producing and memory phase. Given the many abnormalities in the T cell compartment of patients with SLE including an increased activation marker pattern reminiscent of memory cells (Kammer et al., Arthritis Rheum, 2002; Konya et al., Curr Opin Rheumatol, 2014; Moulton and Tsokos, Arthritis Res Ther, 2011), we suggest that the positive correlation of IL-10-producing T cells and disease activity is due to a higher rate/number of activated Th1 cells in peripheral blood of those patients.

We have included this suggestion into the Discussion of the revised manuscript. It should also be noted that there may be a difference in blood-circulating vs. (inflamed) tissue-resident T cell 'behaviour' in these patients, for example, in regards to the kinetics of cytokine production, survival, etc. Thus, due to the lack of an in vivo animal model that would allow us to carefully dissect the role of the novel CD46-MMP9 axis on T and other immune cells in health and disease, our current explanation remains, at this point, speculative and will need to be validated in the future – which we have also made clear in the Discussion.

2. CD46 MFI is strongly reduced in both HC and SLE patients when cells are stimulated by anti-CD3 + anti-CD46 (figure 4A). This data is very likely an artifact due to CD46 ligation by the stimulating anti-CD46 antibody used in cell culture. mRNA evaluation of CD46 should clarify this point.

The rapid down-regulation of surface CD46 protein (by protease shedding and/or internalization) - after

ligand binding including C3b, CD46-binding pathogens or anti-CD46 antibodies - has been observed by several groups on all cell populations assessed so far and is considered as an acknowledged and established 'event' in the field (Gill et al., JEM, 2003; Lovkvist et al., Infect Immun, 2008; Mahtout et al., Oral Microbiol Immunol, 2009; Naniche et al., Virology, 1993; Sakurai et al., Gene Ther, 2007). Interestingly, the functional/biological reason for this observation is not clear for all cells, but in collaboration with Susan Lea and Penny Handford from Oxford University we had recently delivered a potential explanation as to why this may happen on T cells: We had found that on RESTING CD4+ T cells, CD46 binds the Notch-ligand Jagged1 with higher affinity compared to the Notch1-Jagged1 interaction and (Le Friec et al., Nat Immunol, 2012) via this functions as a 'brake/inhibitor'. TCR-induced C3b generation during T cell activation then induces 'autocrine' CD46 down-regulation and via this allows for a productive and Notch1-Jagged1 interaction that is also required for normal Th1 induction – see Supplementary Figure 2 of this manuscript (Cordle et al., Nat Struct Mol Biol, 2008).
As this question raised by Reviewer 1 was also a concern for Reviewer 2 (please see Point 4 below), we would like to also add more technical detail here to demonstrate further that the reduction of CD46 surface expression upon CD46 engagement is indeed correctly reflected by the FACS data shown in Figure 4A in this manuscript:

A. The anti-CD3 and -CD46 antibodies used for T cell activation had been coated onto the plates and non-bound antibody was washed away before cells were added for culture. Thus, there should be no 'free-floating' antibody to interfere with subsequent CD46 measurements (either on the cell surface or the sCD46 generated upon T cell activation).

B. The CD46-activating antibody used coat the culture plates is clone TRA-2-10, which binds to CCPs 1 and 2 of CD46. The Antibody used to subsequently detect CD46 surface expression on resting and activated T cells is clone MEM-258 which recognizes CCP4 in CD46. Neither antibody interferes with each others binding to their respective epitope within CD46.

C. We have performed a set of experiments that combines the kinetics of CD46 cell surface expression by FACS in CD3+CD46-activated T cells with a parallel Western blot analysis of sCD46 accumulating in the cell supernatants. These data also strongly support the reduction of CD46 tethered to the cell surface by cleavage (Figure 2, below). These data also support published work suggesting that CD46 expression 're-appears' by about days 3-4 post T cell activation (Buettner et al., Cancer Res, 2005).

European Journal of Immunology

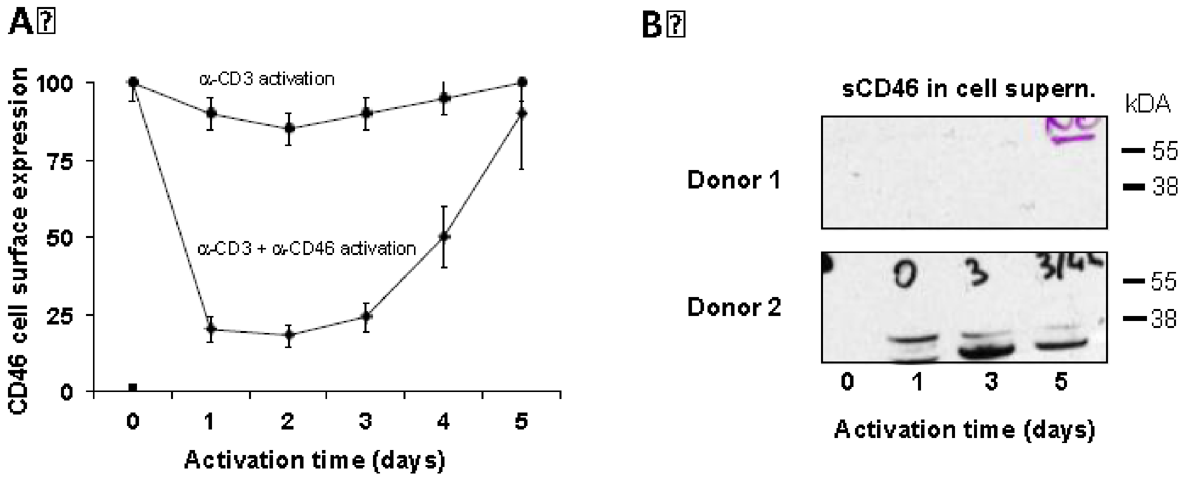

Figure 2. Kinetics of CD46 surface down-regulation/surface cleavage in activated CD4⁺ T cells. (A) CD46 cell surface expression levels on CD3- or CD3+CD46-activated T cells assessed by FACS at indicated time points. Activating Ab to CD46 was TRA-2-10 (recognizing CCPs 1 and 2 within CD46) and the Ab used for cell surface detection of CD46 was MEM258 (recognizing CCP4). Data are derived from n = 3 using a different donor each time. (B) Cleaved CD46 levels released into the cell supernatant as assessed via Western blotting. Purified T cells were CD3+CD46-activated and cell supernatants harvested at the indicated time points. Cell culture media was replaced with fresh media each day. Cell supernatants were concentrated using spin columns and sCD46 detected using gel electrophoresis and Western blotting utilizing a rabbit polyclonal antiserum to human CD46. Note that sCD46 was barely detectable in the supernatants of CD3-activated cells (data not shown).

D. Finally, and as suggested by the Reviewer, a detailed analysis of the mRNA expression profile of all CD46 isoforms present in resting and activated CD4+ T cells has indeed previously been performed by our lab (Kolev et al, Immunity, 2015) (Figure 3, below). These data demonstrated that also activated T cells produce CD46 mRNA but, interestingly, change the levels of mRNAs coding for distinct CD46 isoforms. The mechanism that regulates CD46 mRNA translation and/or (re)appearance of CD46 protein on the T cell surface (please see Figure 1 above) is currently a subject of research in our laboratory.

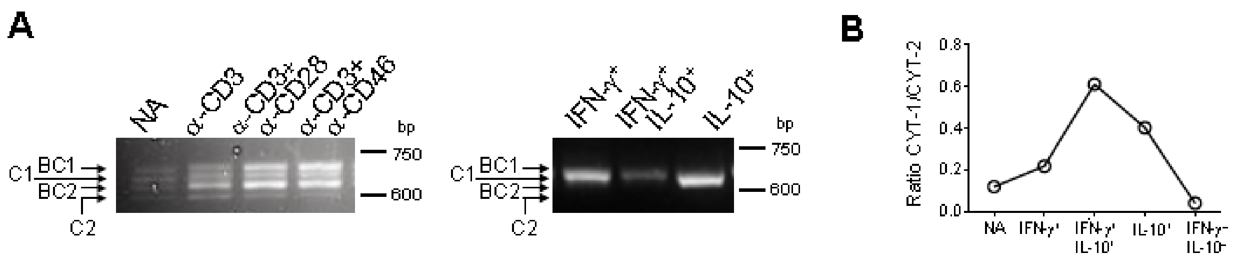

Figure 3. Switches in CD46 isoform-expression correlate with expected metabolic changes during the Th1 life cycle. CD46 isoform mRNA levels in (A) non-activated (NA) and activated T cells (36 h, left panel), and in sorted IFN-γ⁺, IFN-γ⁺IL-10⁺ and IL-10⁺ Th1 subpopulations (activated for 36 h, right panel), and (B) ratio of CYT-1 to CYT-2 tail mRNA expression. Data are derived from n = 3 using a different donor each time. ***Taken from Kolev et al., Immunity 2015.***

European Journal of
Immunology

3. The authors show a direct correlation between IL-10 producing cells and the amounts of sCD46 (figure 4D), this result is in contrast with what expected and with the other data of the work, (i.e. figure 4 E and F). Since SLE patients are composed by "IL-10 switching" and "not switching" subjects (fig 4E), could the correlation be affected by a non-homogeneous group of patients?
The x-axis in Figure 4D shows the ratio between IL-10+ cells after CD3 stimulation divided by IL-10+ cells after CD3 + CD46 stimulation. This ratio is plotted against the amount of sCD46 (y-axis). This translates into: Low values = "switching", high values = "non-switching" and is consistent with the other data of the work. We do apologies for the confusion caused by the misleading labeling of the x-axis and have now changed it accordingly to: "Ratio IL-10+ cells CD3 versus CD3+CD46 " in the revised version of the manuscript and included a better explanation into the figure legend.

4. IL-17 strongly induces MMP9, did the author evaluated the production of IL-17 by T cells from SLE patients?
To address the reviewer's question, we performed additional cytokine analyses on CD4+ T cell supernatants of patients with SLE and healthy controls stimulated with mAb to CD3 alone or co-stimulated with mAb to CD3+CD46. CD46-costimulation induced a significant increase in IL-17 production in T cells from HC, while such increase was not observed in T cells from patients with SLE. Thus, increased production of IL-17 by 'SLE T cells' seems not to be the underlying mechanism of augmented MMP9 production and sCD46 generation. There data are now included in the new Supplementary Figure 2 of the revised manuscript.

Minor points:
1. P values are not reported in figure 3 D
We apologize for this omission and have now added the p values in Figure 3D in the revised version of the manuscript.
2. In figure 3 legend the last sentence starts with "(C)" instead of "(D)"
We apologize for the mistake and changed the legend in Figure 3 accordingly in the revised version of the manuscript.
3. MMP9 inhibitor has opposite effects with regard to IFN-gamma production in HC and in SLE patients (figure 5B and 5E). The author should speculate on some possible explanation.
Yes, this is indeed correct. The inhibition of 'normal' amounts of shedding of CD46 from the surface of T cells from healthy donors, reduces both, IFN-□ and IL-10 production proportionally. The likely reason is that surface CD46 functions as a brake for the Notch1-Jagged1 cis interaction needed for normal Th1 induction in humans and mice (Le Friec et al., Nat Immunol, 2012). Thus, reduced CD46 cleavage/disappearance leads to reduced Notch1-Jagged-1 interactions and reduced IFN-□/Th1 induction. In humans, IL-10 co-production and switching by Th1 cells is dependent on initial IFN-□ secretion (Le Friec et al., Nat. Immunol. 2012; Kolev et al., Immunity, 2013) explaining why IL-10 levels

European Journal of Immunology

are also concomitantly reduced.
The inhibition of CD46 shedding by T cells from patients with SLE, on the other hand, reduces IFN-□ levels but increases IL-10 levels – thus, re-instates normal 'switching'. Figure 4A demonstrates that although CD46 surface levels are downregulated/cleaved normally by T cells from SLE patients (and hence allow for appropriate Notch1-Jagged1 interactions and IFN-□ production) their T cells produce much larger amounts of sCD46 (Figure 4C). Augmented sCD46 levels, on the other hand, inhibit IL-10 switching (Figure 5E in this manuscript and Le Friec et al., Nat Immunol, 2012). Thus, one reason for the observed effects of the MMP9 inhibitor on SLE patients' T cells cells could be that the inhibitor reduces the abnormally high levels of sCD46 in these cultures to levels allowing for normal IL-10 switching (which goes together with reductions in IFN-□ secretion). We have explained this now better and added a pertinent section to the Result part of the revised manuscript.
4. In Figure 5B a p value is reported, while in the text it results that the differences are not significant.
We apologize for this omission and have added the missing p value to Figure 5B in the revised version of the manuscript.

REVIEWER 2:
The present manuscript by Ellinghaus et al. entitled „Dysregulated CD46 sheddig interferes with Th1-contraction in systemic lupus erythemtosus" investigated the pathological role of CD46 and its regulatory pathway in patients with SLE. The authors hypothesized that the CD46 pathway plays a pivotal role in the activation of the Th1 response. Therefore the authors included 45 SLE patients and 38 healthy volunteers. First IFN-γ and IL-10 secretion in isloated CD4+ cells was performed with anti-CD3 and anti-CD46 stimulation resulting in predominantely Th1 response. This pattern seems to be stable over a period of several weeks demonstrated in a rather small subgroup of patients analysed at two visits. The decreased amount of IL-10 production leads to reduced capacity to inhibit B- and T-cells proliferation which has been elegantly assed in two different proliferation tests.
To test whether shedding of sCD46 is the underlying mechanisms of the Th1/IL-10 imbalance the authors studied CD46 expression in resting and activated cells and sCD46 in supernatants. The author concluded that increased CD46 shedding upon activation and increased sCD46 prevent Th1 contraction in vitro. Last the authors showed an effect of MMP-9 inhibition which might indicate that MMP-9 is a crucial factor in mediating CD46 shedding.

The present manuscript deals with an important topic and the data are sound. The introduction is well written. The patients and methods seem to be adequate.
We thank the reviewer for her/his positive remarks and succinct summary and interpretation of our study on the role of MMP-9-generated sCD46 in autocrine Th1 response regulation.
Some minor comments should be addressed:

European Journal of Immunology

1. SLEDAI was used as stated in the results. This should be mentioned in the method part.
We thank the Reviewer for his/her comment and have now mentioned the measurement of disease activity via the SLEDAI score into the Method part of the revised manuscript.

2. Were the patient on immunosuppressive drugs? Which were used?
The following details have been added to materials and methods. “All the patients were treated with hydroxychloroquine, some were also on low dose prednisolone and/or mycophenolate. No patients were on high dose corticosteroids or had ever received anti-CD20 therapy.”

3. The detection levels oft he ELISA should be mentioned in the method part.
We thank the Reviewer for his/her comment and have now included the detection levels of all ELISAs utilized in the Method part of the revised manuscript.

4. Figure 1B and 2A have Total cyt+ cell (%) on the y-axis. What does cyt+ mean? Should be clarified.
The abbreviation ‘cyt+’ stands for ‘cytokine-positive’ cells and the particular cytokine assessed is then depicted on the x-axis. We do agree that this is confusing and have now changed the label of the y-axis from ‘cyt+’ cells to ‘Cytokine positive’ cells and explained further in the figured legend that this refers to ALL IFN-□ or IL-10 positive cells (thus, including the IFN-□+/IL-10- and IFN-□+/IL-10+ cells for the IFN-□-positive cells and IFN-□+/IL-10+ and IFN-□/IL-10+ cells for IL-10-producing cells) in the revised version of the manuscript.

5. Perhaps I do understand it not correct but to my knowledge Figure 4A shows CD46 expression after stimulation with CD3 and after stimulation with anti-CD3 and anti-CD46. To me ist not surprising that anti-CD46 stimulation decreases CD46 expression because the receptor is blocked? Are you sure that you do not detect sCD46 which might be partially the anti-CD46 you added as stimuli? (Figure 4C).
As Reviewer 1 raised a similar concern, please see our response under Point 2 in our response to Reviewer 1.
Beside the above mentioned comments the present manuscript addresses an clinical interesting topic. The presentation of the data is clear. The supplementary figure 2 (now 3) is very helpful.

Second Editorial Decision - 24-Mar-2017

Dear Prof. Vyse, dear Prof. Kemper,

It is a pleasure to provisionally accept your manuscript entitled "Dysregulated CD46 shedding interferes

European Journal of Immunology

with Th1-contraction
in systemic lupus erythematosus" for publication in the European Journal of Immunology. For final acceptance, please follow the instructions below and return the requested items as soon as possible as we cannot process your manuscript further until all items listed below are dealt with.

Please note that EJI articles are now published online a few days after final acceptance (see Accepted Articles: http://onlinelibrary.wiley.com/journal/10.1002/(ISSN)1521-4141/accepted). The files used for the Accepted Articles are the final files and information supplied by you in Manuscript Central. You should therefore check that all the information (including author names) is correct as changes will NOT be permitted until the proofs stage.

We look forward to hearing from you and thank you for submitting your manuscript to the European Journal of Immunology.

Yours sincerely,
Nadja Bakocevic

on behalf of Prof. Francesco Annunziato

Dr. Nadja Bakocevic
Editorial Office
European Journal of Immunology
e-mail: ejied@wiley.com
www.eji-journal.eu
